# Supplementary material for: Content-rich biological network constructed by mining PubMed abstracts
Source: BMC Bioinformatics. 2004 Oct 8;5:147. doi: 10.1186/1471-2105-5-147 (PMC528731; doi:10.1186/1471-2105-5-147)
Supplement: Additional File 5 — The original Chilibot query results of the term "long-term potentiation (LTP)" and 22 other terms, limiting the latest references analyzed to the years 1990, 1995, 2000, and 2004. [file 1471-2105-5-147-S5.bz2 › chilibotAdditionalFile5/ltp2004/html/LTP_TRKB.html]

 


 **LTP** and **TRKB** 
  
Found 34 abstracts in PubMed,  **30 abstracts were retrieved and analyzed**.  


---

 Search Google  |
 PDF files only 
|  EDU domain only 

---

**Interactive relationship** (e.g. stimulation, inhibition, etc)

- Any of several signaling pathways activated by  **trkB**  could be required for  **LTP** .  Ref: 12689763 Trends Neurosci, 2003
- The  **TrkB**  Ab treated slices showed significant deficits in  **LTP**  induced by either theta burst stimulation TBS or pairing.  Ref: 9331355 Neuron, 1997
- The application of a  **TrkB**  IgG 30 min after  **LTP**  induction caused previously potentiated synaptic transmission to return to baseline levels, indicating that  **TrkB**  ligands are required to maintain  **LTP**  for up to 1 hr after induction.  Ref: 9331355 Neuron, 1997
- Activation of  **trkB**  also modulates hippocampal long term potentiation  [ **LTP** ] , in which postsynaptic N methyl D aspartate glutamate receptors play a key role.  Ref: 9223337 Proc Natl Acad Sci U S A, 1997
- Long term potentiation  [ **LTP** ]  and spatial learning are associated with increased phosphorylation of  **TrkB**  and extracellular signal regulated kinase ERK in the dentate gyrus evidence for a role for brain derived neurotrophic factor.  Ref: 12049326 Behav Neurosci, 2002
- **TrkB**  reduction affects presynaptic function and reduces the ability of tetanic stimulation to induce  **LTP** .  Ref: 10995833 J Neurosci, 2000
- In the Schaffer collateral pathway of the hippocampus, binding of BDNF to high affinity  **trkB**  receptors is essential for the induction of long term potentiation  [ **LTP** ] , a specific type of synaptic plasticity.  Ref: 12628355 Trends Pharmacol Sci, 2003
- Incubation of hippocampal slices with  **TrkB**  Ab had no effect on basal synaptic transmission, short term plasticity, or  **LTP**  induced by several trains of tetanic stimulation.  Ref: 9331355 Neuron, 1997
- The present results demonstrate that Trk B  [ **TRKB** ]  activation is required for the long term maintenance, > 30 min, of both  **LTP**  and  **LTP**  after LTD at P17.  Ref: 10762369 Eur J Neurosci, 2000
- Brain derived neurotrophic factor and its receptor,  **trkB** , activate cAMP response element binding protein and the transcription of genes that is crucial for long term potentiation  [ **LTP** ] .  Ref: 12689763 Trends Neurosci, 2003
- Here we attempt to address this question by investigating, first, its release following induction of  **LTP**  in perforant path granule cell synapses and, second, the signalling events which follow activation of the BDNF receptor,  **TrkB** , in the presynaptic terminal.  Ref: 11389170 J Neurochem, 2001
- We found that hippocampal long term potentiation  [ **LTP** ]  was impaired in  **trkB**  mice, but not  **trkB**  mice.  Ref: 12367511 Neuron, 2002
- At P23, a higher concentration of  **TrkB**  IgG was necessary to impair  **LTP** .  Ref: 10762369 Eur J Neurosci, 2000
- Consistent with this, elimination of  **TrkB**  in postsynaptic neurons does not affect  **LTP** .  Ref: 10995833 J Neurosci, 2000
- Shc binding site in the  **TrkB**  receptor is not required for hippocampal long term potentiation  [ **LTP** ] .  Ref: 10699438 Neuropharmacology, 2000
- The late phase of  **LTP**  2 3 hr was also significantly impaired in slices pretreated with  **TrkB**  Ab or a  **TrkB**  IgG.  Ref: 9331355 Neuron, 1997
- However, both  **TrkB**  IgG fusion proteins and BDNF antibody similarly reduced  **LTP** , suggesting that only BDNF but no other ligands of the  **TrkB**  receptor are likely to be involved in  **LTP**  induction.  Ref: 10479698 J Neurosci, 1999
- Taken together, these results indicate that endogenous Trk B  [ **TRKB** ]  ligands are necessary for the expression of  **LTP**  but not LTD at a critical time during postnatal cortical development.  Ref: 10762369 Eur J Neurosci, 2000
- This work has identified particular ITFs and neurotrophin systems tyrosine receptor kinase B, Trk B  [ **TRKB** ]  system that may be involved in stabilizing long lasting  **LTP** .  Ref: 10080384 Prog Neurobiol, 1999
- Brain derived neurotrophic factor BDNF and its receptor, tyrosine receptor kinase trk B  [ **TRKB** ] , play important roles in neural plasticity, long term potentiation  [ **LTP** ]  and memory formation.  Ref: 12473084 Eur J Neurosci, 2002
- Inhibition of p75NTR does not detectably reduce  **LTP**  or affect presynaptic function, but analyses of newly generated  **trkB**  mutants implicate  **TrkB** .  Ref: 10995833 J Neurosci, 2000
- **TrkB**  IgG or K252a itself completely blocked  **LTP** , suggesting that endogenous BDNF or another  **TrkB**  ligand plays a role in  **LTP**  in the developing visual cortex.  Ref: 9254683 J Neurosci, 1997
- Moreover, normal  **LTP**  is generated in the mutant with reduced  **TrkB**  by a depolarization low frequency stimulation pairing protocol that puts minimal demands on presynaptic terminal function.  Ref: 10995833 J Neurosci, 2000

**Parallel relationship** (e.g. studied together, co-existance, homology, etc.)

- Slices from BDNF knock out animals or slices treated with  **TrkB**  antibodies failed to exhibit L  **LTP** , indicating that  **TrkB**  ligands participate in extending synaptic enhancement from a short lasting to a long lasting form.  Ref: 9704996 NeuropharmacologyNeuropharmacology, 2000
- To test whether the Shc site activated signaling pathway, which has been shown to be important for neuronal survival in vivo, also plays a role in processes like long term potentiation  [ **LTP** ]   **LTP** , we have generated a mouse strain carrying a mutation in the Shc binding site of the  **TrkB**  receptor.  Ref: 10699438 Neuropharmacology, 2000
- We examined the role of  **TrkB**  ligands in hippocampal long term potentiation  [ **LTP** ]   **LTP**  using function blocking  **TrkB**  antiserum Ab and Trk IgG fusion proteins.  Ref: 9331355 Neuron, 1997
- We have examined whether BDNF or other  **TrkB**  ligands participate in L  **LTP**  in two ways by examining transgenic mice which lack BDNF or by acutely blocking  **TrkB**  function using function blocking antibodies.  Ref: 9704996 NeuropharmacologyNeuropharmacology, 1997
- A recent study using mice carrying a mutation in a specific tyrosine residue of  **trkB**  pinpoints a necessary role for the phospholipase Cgamma pathway in CREB activation and  **LTP**  maintenance.  Ref: 12689763 Trends Neurosci, 2003
- Long term potentiation  [ **LTP** ]  in the dentate gyrus of the rat hippocampus is accompanied by brain derived neurotrophic factor induced activation of  **TrkB** .  Ref: 11389170 J Neurochem, 2001
- Thus, BDNF appears to act through  **TrkB**  presynaptically... to modulate  **LTP** .  Ref: 10995833 J Neurosci, 2000
- Because the reagents used in acute experiments react not only with BDNF but also with neurotrophin 4 5 NT4 5 and neurotrophin 3 NT3, we examined the involvement of these neurotrophins in  **LTP**  using two highly specific, function blocking monoclonal antibodies against BDNF and NT3, as well as a  **TrkB**  IgG fusion protein.  Ref: 10479698 J Neurosci, 1999
- Slices exposed to the same number of inducing stimuli, delivered either as TBS or as a single 100 Hz epoch, only exhibited  **TrkB**  sensitive  **LTP**  when TBS was used, indicating that the temporal pattern of stimulation determines the neurotrophin dependence.  Ref: 9331355 Neuron, 1997
- Mechanism of  **TrkB**  mediated hippocampal long term potentiation  [ **LTP** ] .The  **TrkB**  receptor tyrosine kinase and its ligand, BDNF, have an essential role in certain forms of synaptic plasticity.  Ref: 12367511 Neuron, 2002
- We found that homo and heterozygous mutant mice show no difference in the induction rate or magnitude of E  **LTP**  and L  **LTP**  induced by theta burst or tetanus stimulation, suggesting that the Shc binding site in the  **TrkB**  receptor and its downstream activated signaling cascade is not involved in hippocampal synaptic plasticity.  Ref: 10699438 Neuropharmacology, 2000
- Trk B  [ **TRKB** ]  signalling controls  **LTP**  but not LTD expression in the developing rat visual cortex.  Ref: 10762369 Eur J Neurosci, 2000
- Here, we examined the contribution of endogenous Trk B  [ **TRKB** ]  activation to the regulation of different forms of synaptic plasticity including long term potentiation  [ **LTP** ]   **LTP** , long term depression LTD and  **LTP**  after LTD in the developing visual cortex.  Ref: 10762369 Eur J Neurosci, 2000
- showing brain derived neurotropic factor modulates  **LTP**  by binding to  **TrkB**  receptors on the postsynaptic neuron.  Ref: 11872822 Science, 2002
- The role of brain derived neurotrophic factor receptors in the mature hippocampus modulation of long term potentiation  [ **LTP** ]  through a presynaptic mechanism involving  **TrkB** .  Ref: 10995833 J Neurosci, 2000
- Brain derived neurotrophic factor BDNF and its receptor  **TrkB**  regulate both short term synaptic functions and long term potentiation  [ **LTP** ]   **LTP**  of brain synapses, raising the possibility that BDNF  **TrkB**  may be involved in cognitive functions.  Ref: 10571233 Neuron, 1999
- Neurotrophins and time different roles for  **TrkB**  signaling in hippocampal long term potentiation  [ **LTP** ] .  Ref: 9331355 Neuron, 1997
